# Metabolic engineering strategies for de novo biosynthesis of sterols and steroids in yeast

**DOI:** 10.1186/s40643-021-00460-9

**Published:** 2021-11-05

**Authors:** Yuehao Gu, Xue Jiao, Lidan Ye, Hongwei Yu

**Affiliations:** 1 Key Laboratory of Biomass Chemical Engineering (Education Ministry), College of Chemical and Biological Engineering, Zhejiang University, Hangzhou, 310027 China; 2 Institute of Bioengineering, College of Chemical and Biological Engineering, Zhejiang University, Hangzhou, 310027 China

**Keywords:** Sterols and steroids, De novo biosynthesis, Yeast, Metabolic engineering

## Abstract

Steroidal compounds are of great interest in the pharmaceutical field, with steroidal drugs as the second largest category of medicine in the world. Advances in synthetic biology and metabolic engineering have enabled de novo biosynthesis of sterols and steroids in yeast, which is a green and safe production route for these valuable steroidal compounds. In this review, we summarize the metabolic engineering strategies developed and employed for improving the de novo biosynthesis of sterols and steroids in yeast based on the regulation mechanisms, and introduce the recent progresses in de novo synthesis of some typical sterols and steroids in yeast. The remaining challenges and future perspectives are also discussed.

## Introduction

**Fig. 1:**
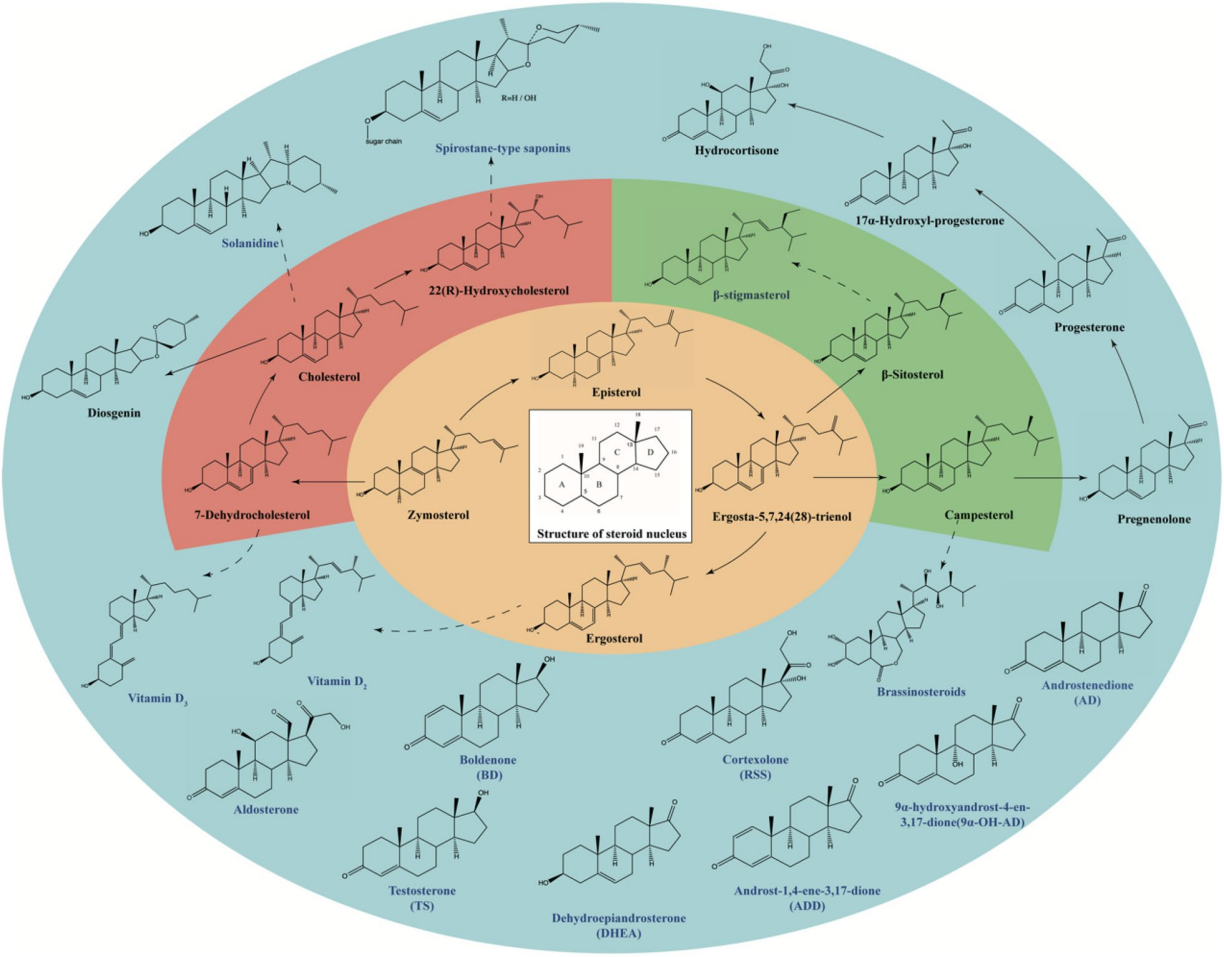
Examples of sterols and steroids. The endogenous sterol pathway in yeast is highlighted in yellow, heterogenous synthesis of animal-derived sterols in yeast is highlighted in red, heterogenous synthesis of phytosterols in yeast is highlighted in green and steroids derived from sterols are highlighted in blue. Solid arrows represent de novo routes examined in yeast and dashed arrows indicate potential synthetic pathways. De novo biosynthesis of the steroidal compounds marked in blue are not yet realized in yeast

Steroidal compounds are specific polycyclic terpenoids identified by a carbon skeleton called *perhydrocyclopentanophenanthrene* (Tantuco et al. 2000), with sterols and steroids which can be further divided into steroidal hormones, steroidal saponins and steroidal alkaloids as typical examples (Fig. 1). Sterols and steroids are extremely valuable in the pharmaceutical field. Ergosterol and 7-dehydrocholesterol (7-DHC) as typical sterols are important precursors of vitamin D_2_ and vitamin D_3_, respectively (Bikle 2014; Tan et al. 2003). Steroidal saponins such as diosgenin are mainly used for synthesis of steroidal drugs (Wang et al. 2007), while steroidal alkaloids possess potential antimicrobial, analgesic, anticancer and anti-inflammatory effects (Dey et al. 2019). Steroidal hormone drugs are the second largest category of medicine in the world, right after the antibiotics, and the global market exceeds 10 billion dollars (Fernandez-Cabezon et al. 2018).

The natural sources of sterols and steroids are animals and plants, extraction from which is costly due to the limited contents. Chemical synthesis of sterols and steroids from simple molecules has been developed (Kovganko and Ananich 1999; Nemoto et al. 1986), but the lengthy synthetic route and poor yields are obstacles to industrialization. Manufacturing steroids from natural substrates with the basic steroidal nucleus, like cholesterol, phytosterols and tigogenin, by semi-synthetic modification has also been realized (Ohta et al. 1997; Sambyal and Singh 2020). With rapid development of synthetic biology, de novo biosynthesis of steroids from simple carbon sources like glucose using engineered microbial cell factories has emerged as a promising alternative approach.

*Saccharomyces cerevisiae* has been extensively employed as the chassis organism for steroids biosynthesis since it is generally regarded as safe (GRAS) feature, well-studied genetic background and readily available manipulation tools. Up to now, de novo synthesis of cholesterol (Souza et al. 2011), phytosterols (Xu et al. 2020), diosgenin (Cheng et al. 2021), hydrocortisone (Szczebara et al. 2003), pregnenolone (Duport et al. 1998) has been enabled in *S. cerevisiae*. Besides, non-conventional yeast like *Yarrowia lipolytica* and *Pichia pastoris* have also been engineered to produce some sterols and steroids, like pregnenolone and cholesterol (Hirz et al. 2013; Zhang et al. 2019). Recently, the advances on steroid bioproduction in yeast through biotransformation have been well reviewed together with the construction of de novo synthesis pathways for steroids without C24‑alkyl and steroids with saturated C7–C8 bond (Xu and Li 2020). In the present review, we will focus on the regulatory principles and metabolic engineering strategies for improving the de novo production of sterols and steroids in yeast, and introduce the recent progresses in de novo biosynthesis of typical sterols and steroids. The existing challenges and future perspectives are also discussed.

## The regulation mechanisms of sterols and steroids biosynthesis in yeast

Ergosterol synthesis pathway is the natural sterol synthetic pathway in yeast and shares a number of intermediates with the biosynthesis of cholesterol and phytosterols such as zymosterol (Souza et al. 2011) and episterol (Xu et al. 2020). Therefore, yeast is a suitable host for production of cholesterol and phytosterols, and can be further engineered for de novo synthesis of other valuable steroids (Xu and Li 2020). However, the biosynthesis efficiency of heterogenous sterols and steroids is limited by the native regulation network in yeast, including the competition between endogenous pathways and heterogenous enzymes (Guo et al. 2018), the rate-limiting enzymes in the shared precursor pathway for ergosterol and the target sterols and steroids (Veen et al. 2003), the mechanism of sterol homeostasis (Wriessnegger and Pichler 2013), and other linked metabolic pathways.

### Regulation of the ergosterol synthesis pathway

**Fig. 2:**
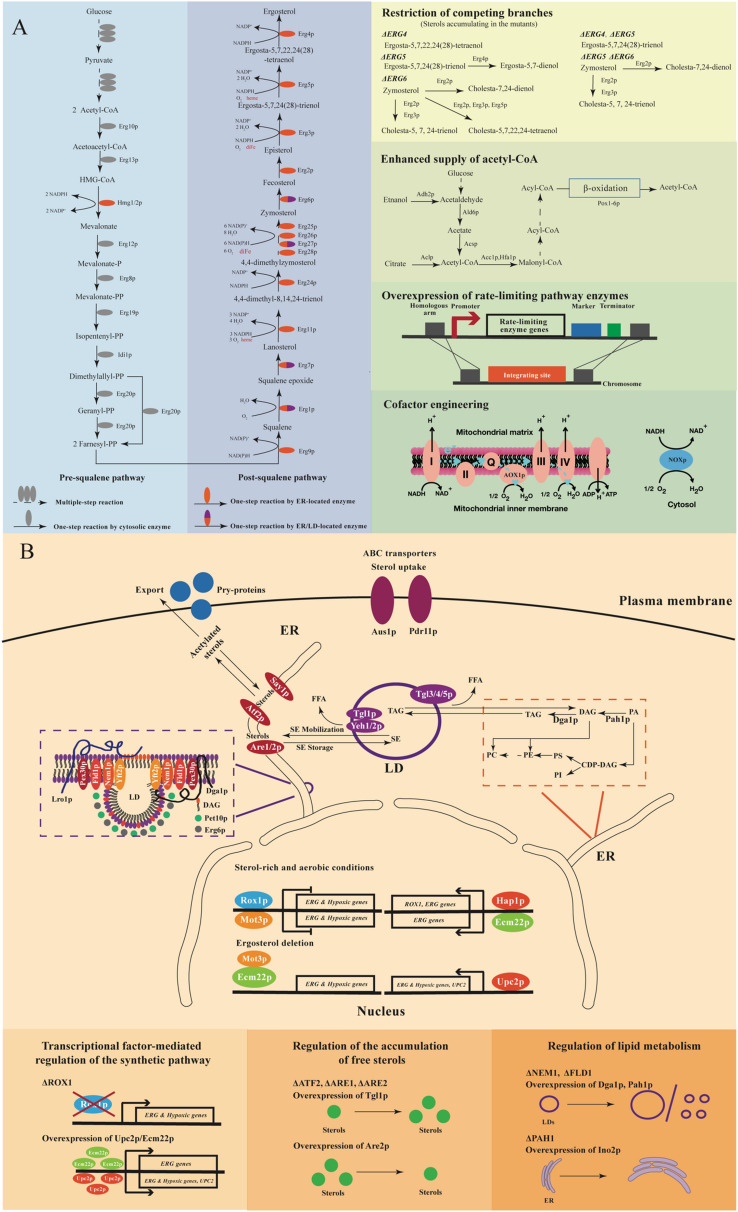
Regulation mechanisms and the corresponding metabolic engineering strategies for regulating de novo biosynthesis of sterols and steroids in yeast. **A** Mechanism and strategies for regulation of the ergosterol synthesis pathway; **B** Mechanism and strategies for regulation of sterol homeostasis. Enzymes: *CoA* coenzyme A; *Erg10p* acetoacetyl-CoA thiolase; *Erg13p* hydroxymethylglutaryl-coenzyme A synthase; *Hmg1/2p* hydroxymethylglutaryl-coenzyme A reductase; *Erg12p* mevalonate kinase; *Erg8p* phosphomevalonate kinase; *Erg19p* diphosphomevalonate decarboxylase; *Idi1p* isopentenyl diphosphate isomerase; *Erg20p* polyprenyl synthetase; *Erg9p* squalene synthetase; *Erg1p* squalene epoxidase; *Erg7p* lanosterol cyclase/lanosterol synthase; *Erg11p* cytochrome P450 lanosterol 14α-demethylase; *Erg24p* sterol C-14 reductase; *Erg25p* C-4 methyl sterol oxidase; *Erg26p* sterol C-4 decarboxylases; *Erg27p* 3-keto-steroid reductase; *Erg6p* C-24 sterol methyltransferase; *Erg2p* C-8 sterol isomerase; *Erg3p* C-5 sterol desaturase; *Erg5p* C-22 sterol desaturase; *Erg4p* C24 (28) sterol reductase; *Adh2p* alcohol dehydrogenase; *Ald6p* aldehyde dehydrogenase; *Acsp* acetyl-CoA synthetase; *Aclp* ATP-citrate lyase; *Acc1p*/*Hfa1p* acetyl-CoA carboxylase; *Pox1*-*6p* peroxisome acyl-CoA oxidase 1–6; *AOX1p* alternative oxidase; *NOXp* NADH oxidase; *Are1*/*2p* acyl-CoA sterol acyltransferase; *Tgl1p* steryl ester hydrolase; *Yeh1*/*2p* yeast steryl ester hydrolase; *Atf2p* acetyltransferase; *Say1p* steryl acetyl hydrolase; *Tgl3*/*4*/*5p* triacylglycerol lipase; *Dga1p* diacylglycerol acyltransferase; *Pah1p* phosphatidic acid phosphohydrolase; *Lro1p* triacylglycerol synthase; *Fld1p* seipin involved in LD assembly; *Pex30p* peroxisome related protein; *Pet10p* perilipin involved in formation and stability of LDs; *Aus1p* ABC protein involved in uptake of sterols; *Pdr11p* pleiotropic drug resistance protein 11. Substrates: *HMG*-*CoA* 3-hydroxy-3-methylglutaryl-CoA; *P* phosphate; *PA* phosphatidic acid; *DAG* diacylglycerol; *TAG* triacylglycerol; *SE* steryl ester; *FFA* free fatty acids; *CDP*-*DAG* cytidine diphosphate-diacylglycerol; *PI* phosphatidylinositol; *PS* phosphatidylserine; *PE* phosphatidylethanolamine; *PC* phosphatidylcholine

Ergosterol synthesis in yeast has been extensively studied, and can be divided into three stages: mevalonate biosynthesis, farnesyl pyrophosphate biosynthesis, and ergosterol biosynthesis [as reviewed in Liu et al. (2019)]. Since excessive accumulation of squalene is often observed in biosynthesis of sterols and steroids (Polakowski et al. 1998), the synthesis pathway can also be decomposed into the pre-squalene synthesis pathway and the post-squalene synthesis pathway (Fig. 2A). The flux of the pre-squalene synthesis pathway relies largely on the mevalonate (MVA) pathway, with 3-hydroxy-3-methylglutaryl-CoA reductase (Hmgrp) as the main rate-limiting enzyme (Hu et al. 2017). Hmgrp degradation via the ER-related degradation (ERAD) pathway acts as a native regulation mechanism for maintaining sterol homeostasis in case of excessive sterols (Jorda and Puig 2020), while Hmgrp overexpression is a common metabolic engineering strategy for enhancing sterols production. Besides, as the starting material of the MVA pathway, supply of acetyl coenzyme A (acetyl-CoA) also regulates the pre-squalene pathway flux (Su et al. 2015). In the post-squalene pathway, the conversion of squalene to squalene epoxide catalyzed by squalene epoxidase (Erg1p) is a major rate-limiting step and its activity is restricted by oxygen availability (Jandrositz et al. 1991). The catalytic reactions of cytochrome P450 lanosterol 14α-demethylase (Erg11p), C-4 methyl sterol oxidase (Erg25p), C-5 sterol desaturase (Erg3p) and C-22 sterol desaturase (Erg5p) also utilize molecular oxygen as the electron acceptor. Besides oxygen, iron is also required in multiple enzymatic steps of the post-squalene pathway. For example, Erg11p and Erg5p as members of the cytochrome P450 family require both oxygen and iron for the synthesis of heme; Erg25p and Erg3p are oxo-diiron enzymes belonging to the fatty acid hydroxylase/sterol desaturase family (Shakoury-Elizeh et al. 2010). Among the metabolic intermediates in the post-squalene pathway, zymosterol is the first found to be able to substitute ergosterol as a yeast membrane component (Zinser et al. 1993). The conversion of zymosterol to ergosterol involves five Erg enzymes [Erg6p, C-24 sterol methyltransferase; Erg2p, C-8 sterol isomerase; Erg3p, C-5 sterol desaturase; Erg5p; Erg4p, C24 (28) sterol reductase], the deletion of which did not affect yeast viability (Abe and Hiraki 2009; Palermo et al. 1997). Previous studies suggested low substrate specificity of these enzymes, which is associated with the generation of various intermediates (Heese-Peck et al. 2002). Therefore, diverse sterols can be accumulated by blocking different genes in *ERG2-6* (Liu et al. 2017). The composition of sterols under different blocking strategies was well summarized in Johnston’s review (Johnston et al. 2020). Modifying the native ergosterol synthesis pathway by combined overexpression of *ERG4* and *ARE2* (encoding acyl-CoA sterol acyltransferase) genes has been shown efficient in improving ergosterol production (He et al. 2007), which also provides hints for construction of yeast cell factories with high production of sterols and steroids.

The enzymes of the post-squalene pathway locate in the endoplasmic reticulum (ER) where sterols synthesis takes place, and some of them [Erg1p, Erg7p (lanosterol cyclase/lanosterol synthase), Erg27p (3-keto-steroid reductase) and Erg6p] can also be found in lipid droplets (LDs) where neutral lipids including steryl esters (SE) and triacylglycerols (TAG) are stored. Intriguingly, Erg6p is mainly found in LDs (Leber et al. 1998), and it is recruited to nascent LDs, contributing to the emergence, growth and stability of LDs (Choudhary and Schneiter 2020). Expansion of the ER by overexpression of the regulator Ino2p was found conductive to the production of squalene and terpenes (Kim et al. 2019). However, this strategy failed to improve the production of 22-hydroxycampest-4-en-3-one, which implied expanding ER was not as efficient for promoting the post-squalene pathway as for the pre-squalene pathway (Xu et al. 2020). In addition, the yeast peroxisome was confirmed to be a storage room and factory for squalene overproduction by compartmentalizing the squalene synthesis pathway in peroxisomes, which may be also applicable for improving the production of sterols and steroids (Liu et al. 2020).

### Regulation of sterol homeostasis

Sterol homeostasis is the endogenous mechanism to adjust membrane components in diverse environments and prevent the accumulation of free sterols which is toxic to yeast (Espenshade and Hughes 2007; Wriessnegger and Pichler 2013). The mechanisms for sterol homeostasis in *S. cerevisiae* (Fig. 2B), including transcriptional factors, feedback inhibition and sterol detoxification, have been well revealed (Jorda and Puig 2020). Expression of Erg enzymes are regulated at the transcriptional level by the sterol regulatory element (SRE)-binding proteins Upc2p and Ecm22p, the heme-binding protein Hap1p and the repressors Rox1p and Mot3p. Sterol synthesis in the pre-squalene pathway is regulated partially by the feedback-regulated degradation of Hmgrp (Gardner et al. 2001). There are four ways to avoid toxification by excessive amounts of free sterols in yeast, including esterification of sterols with fatty acids by the acyl-CoA sterol acyltransferases Are1p and Are2p, downregulation of sterol synthesis, sterol acetylation by Atf2p (acetyltransferase), and secretion by Pry1-3p (Pathogen related yeast protein) (Ploier et al. 2015). When the amount of free sterols is not sufficient, they can be re-mobilized from SE stored in LDs by the function of lipases, mainly Yeh1p (yeast steryl ester hydrolase 1), Yeh2p (yeast steryl ester hydrolase 2) and Tgl1p (steryl ester hydrolase) (Rajakumari et al. 2008; Wagner et al. 2009) and acetylated sterols can be deacetylated by the function of Say1p (steryl deacetylase) (Tiwari et al. 2007). Therefore, LDs play an important role in cellular lipid homeostasis through controlling the metabolic flux as well as the availability of sterols and fatty acids (Kohlwein et al. 2013). Another mechanism of sterol homeostasis is sterol transport which involves many proteins (Jacquier and Schneiter 2012; Wriessnegger and Pichler 2013). For instance, two plasma membrane ABC (ATP-binding cassette) transporters Aus1p and Pdr11p, are involved in the uptake of external sterols under anaerobic conditions to cope with the blocked sterol synthesis (Kohut et al. 2011).

Besides sterols, there are many other important kinds of lipids in yeast such as sphingolipid, phospholipid, fatty acids, and SE. Although researches on these lipids synthesis pathways are always independent from each other, there is a rising body of evidence to reveal tight connections among them, which means the metabolism of these related lipids is also involved in the regulation of sterol homeostasis (Alvarez-Vasquez et al. 2011; Ploier et al. 2015; Shin et al. 2012). The dynamic ergosterol model constructed by the guidelines of Biochemical Systems Theory (BST) has illustrated the functional integration of the yeast sphingolipid–ergosterol (SL-E) pathway (Alvarez-Vasquez et al. 2011). Sterols are transported by non-vesicular mechanisms to the plasma membrane (PM) and a model about ER–PM contact sites has been built to provide a bond for coordinating the complex interrelationship between sterols, sphingolipids, and phospholipids which are the main components of PM (Quon et al. 2018). Besides, there are many evidences to support a metabolic link between the SE metabolism and the biosynthesis of sterols and fatty acids (Ploier et al. 2015). For instance, the total sterols decreased when sterol esterification was blocked by the negative regulation targeting *ERG3* (ArthingtonSkaggs et al. 1996). SE metabolism is also linked with triacylglycerol metabolism by triacylglycerol lipases, mainly Tgl3p, Tgl4p and Tgl5p, which catalyze the degradation of triacylglycerols stored in LDs (Schmidt et al. 2014).

## Metabolic engineering strategies for heterologous production of sterols and steroids in yeast

To enhance the heterologous production of sterols and steroids in yeast, various metabolic engineering strategies have been developed based on the endogenous regulation mechanisms. Typical strategies include regulating the ergosterol synthesis pathway via enhancing the pathway flux by restriction of the competing branches, strengthening precursor supply, overexpression of rate-limiting enzymes, and/or reconstruction of cofactor balance; and regulating sterol homeostasis via deletion or overexpression of the transcriptional factors, regulation of the accumulation of free sterols, and/or regulation of lipid metabolism (Fig. 2).

### Restriction of competing branches

Diverse sterols can be accumulated by knockout of *ERG2-6* which are found as non-essential genes (Johnston et al. 2020) and encode enzymes with broad substrate specificity. *ERG6* encodes C-24 sterol methyltransferase that converts zymosterol to fecosterol (Hu et al. 2017). By disrupting *ERG6*, more cholesta-5,7,24-trienol was formed from zymosterol through the reactions catalyzed by Erg2p and Erg3p, which is the key precursor of 7-DHC and could be transformed into cholesta-5,7,22,24-tetraenol by Erg5p (Heese-Peck et al. 2002). By deletion of *ERG5* and introduction of the heterologous *Gg_DHCR24* gene from *Gallus gallus* encoding Δ^24^‐dehydrocholesterol reductase, a 7-DHC producing yeast SyBE_Sc01250009 was constructed from the original host SyBE_Sc01130007 with a strengthened pre-squalene pathway. Further knockout of *ERG6* as a competing branch led to increased accumulation of zymosterol and thus improved 7-DHC production by 77.6% (Guo et al. 2018). Campesterol is another key intermediate for many valuable steroids. A campesterol-producing *Y. lipolytica* strain was built by disruption of *ERG5* causing the accumulation of ergosta-5,7-dienol together with heterologous expression of the 7-dehydrocholesterol reductase gene (*DHCR7*) from *Xenopus laevis* (Du et al. 2016). By deleting *ERG5* and *ERG4* encoding enzymes for converting ergosta-5,7,24(28)-trienol to ergosta-5,7,22,24(28)-tetraenol and ergosta-5,7-dienol, respectively, the accumulation of ergosta-5,7,24(28)-trienol was enhanced, which contributed to the improved production of 24-methylenecholesterol (Sawai et al. 2014). In addition to restriction of the cross-talk among Erg2-6p, new competitions may occur in the reconstructed metabolic pathway due to the introduction of exogenous enzymes. For example, in the β-sitosterol synthesis pathway constructed by introducing *DWF1* (Δ^24(28)^-sterol reductase), *DWF5* (C7(8)-reductase), *DWF7* (Δ^7^-sterol-C5(6)-desaturase) and *SMT2* (24-methylenesterol C-methyltransferase 2), 24-methylenecholesterol as the substrate of SMT2p was competitively consumed by Erg4p. When *ERG4* was knocked out, the production of β-sitosterol was improved by four times (Xu et al. 2020).

### Enhanced supply of acetyl-CoA

As a key metabolite of carbon and energy metabolism in yeast, acetyl-CoA serves as the starting compound of the mevalonate pathway and is thus an important precursor for sterols biosynthesis. The major source of acetyl-CoA in *S. cerevisiae* is the dehydrogenation of acetaldehyde followed by ligation of acetate and CoA catalyzed by Aldp (acetaldehyde dehydrogenase) and Acsp (acetyl-CoA synthetase) (Saint-Prix et al. 2004; Takahashi et al. 2006). Fatty acid β-oxidation occurring in yeast peroxisomes is another way to generate acetyl-CoA (Chen et al. 2013; Takahashi et al. 2006). In addition, the ATP-dependent citrate lyase (Aclp) naturally present in oleaginous yeast (such as *Y. lipolytica*) uses citric acid as a substrate and converts it to acetyl-CoA and oxaloacetate. When Aclp was heterologously expressed in *S. cerevisiae*, the supply of acetyl-CoA was improved (Lian et al. 2014; Tang et al. 2013). Co-overexpression of *ADH2* (alcohol dehydrogenase 2), *ALD6*, *ACS*^*L641P*^ (from *Salmonella enterica*) and *ACL* (from *Mus musculus*) in *S. cerevisiae* redirected the glycolytic flux to acetyl-CoA and resulted in 64.29% and 41.04% increase of acetyl-CoA accumulation in the mid-logarithmic phase and stationary phase, respectively, and meanwhile the 7-dehydrocholesterol production was improved by 85.44% (Su et al. 2015). When Pox2p (peroxisome acyl-CoA oxidase) with high catalytic activity and specificity for β-oxidation of long-chain fatty acids (Luo et al. 2002; Mlickova et al. 2004) was overexpressed together with Aclp in *Y. lipolytica*, the cytoplastic acetyl-CoA supply was enhanced by simultaneous improvement of citrate cleavage and β-oxidation, leading to elevated production of campesterol (Zhang et al. 2017).

### Overexpression of rate-limiting pathway enzymes

Identification and elimination of rate-limiting reactions in the synthetic pathway is a common and efficient metabolic engineering strategy towards enhanced production of the target metabolite (Paramasivan and Mutturi 2017a). In the pre-squalene pathway, Hmgrp is the main rate-limiting enzyme. By overexpressing tHmg1p in *S. cerevisiae*, large amounts of squalene were accumulated and the levels of ergosterol and other sterol compounds were also slightly increased (Polakowski et al. 1998). In some studies, the complete MVA pathway (Erg10p, acetoacetyl-CoA thiolase; Erg13p, hydroxymethylglutaryl-coenzyme A synthase; tHmg1p; Erg12p, mevalonate kinase; Erg8p, phosphomevalonate kinase; Erg19p, diphosphomevalonate decarboxylase; Idi1p, isopentenyl diphosphate isomerase; Erg20p, polyprenyl synthetase) were overexpressed to improve the production of sterols (Guo et al. 2018; Xu et al. 2020). Overexpression of the enzymes in the pre-squalene pathway increased the content of sterols and meanwhile caused accumulation of squalene, which means improving the conversion from squalene to downstream sterols is crucial for further enhancement of sterols production (Guo et al. 2018; Xu et al. 2020). In the post-squalene pathway, overexpression of *ERG1* led to a significant decrease in squalene accumulation, accompanied by a large increase in lanosterol and a slight increase in later sterols from zymosterol to ergosterol, while overexpression of *ERG11* reduced the accumulation of lanosterol and increased the amounts of the downstream sterols (Veen et al. 2003). Erg4p is known as another rate-limiting enzyme in the sterols synthetic pathway, overexpression of which led to an increase of ergosterol production in *S. cerevisiae* (He et al. 2003). In addition, overexpression of Erg2p and Erg3p from *M. musculus* enhanced the production of 7-DHC in yeast (Lang and Veen 2006). Furthermore, the heterologous enzymes are often rate-limiting, so their copy number should also be considered. The campesterol synthesis in *Y. lipolytica* was boosted by introducing two copies of *DHCR7* (Qian et al. 2020). Similarly, the yield of 7-DHC in *S. cerevisiae* was improved by 16.5% when introducing a second copy of *Gg_DHCR24* from *Gallus gallus* (Guo et al. 2018).

### Cofactor engineering

Redox imbalance is a common issue in strains harboring heterologous pathways, while redox homeostasis plays important roles in cell activities (King and Feist 2014). Therefore, reconstruction of redox balance through cofactor engineering is an important strategy to improve the production of the target metabolites. In *S. cerevisiae*, the intracellular redox potential is mainly determined by the NADH/NAD^+^ ratio and to a lesser extent by the NADPH/NADP^+^ ratio (Vemuri et al. 2007). Studies have found that knocking out *ERG5* caused redox imbalance and the ratio of cytosolic free NADH/NAD^+^ became higher than that of the wild type (Su et al. 2015). By construction of a cofactor regeneration system composed of a water-forming NADH (nicotinamide adenine dinucleotide) oxidase (NOXp) and an alternative oxidase (AOX1p), the ratio of cytosolic free NADH/NAD^+^ was efficiently decreased alleviating the redox imbalance, and the production of 7-DHC by engineered *S. cerevisiae* in shake-flask culture was increased by 74.4% (Su et al. 2015).

NADPH (nicotinamide adenine dinucleotide phosphate) is another important cofactor for oxidoreductases. In the ergosterol synthesis pathway, many enzymes such as tHmg1p and cytochrome P450 enzymes (CYPs) are NADPH-dependent, and 16 molecules of NADPH are demanded for production of one molecule of ergosterol (Hu et al. 2017). In the production of terpenoids in *S. cerevisiae*, improving the supply of NADPH by overexpression of Zwf1p (glucose-6-phosphate dehydrogenase) (Zhao et al. 2015), Stb5p (transcription factor) (Hong et al. 2019), or Pos5p (mitochondrial NADH kinase) (Zhao et al. 2015) increased the yield by 18.8–65.6%. Similarly, co-expression of Pos5p and tHmg1p significantly enhanced the production of squalene (Paramasivan and Mutturi 2017b). In addition, involvement of heterologous CYPs such as CYP11B1p (11β-steroid hydroxylase) in the sterols and steroid pathway indicates requirement of additional redox potential (Szczebara et al. 2003). Therefore, cofactor engineering may be a promising strategy for improving the de novo synthesis of heterologous steroids and sterols, which is, however, seldom reported.

### Transcriptional factor-mediated regulation of the synthetic pathway

There are a set of transcription factor regulation systems that respond to sterol deficiency in *S. cerevisiae*, such as Rox1p, Mot3p, Hap1p, Ecm22p and Upc2p (Davies and Rine 2006; Klinkenberg et al. 2005; Montanes et al. 2011). In sterol-rich and aerobic conditions, the basic expression of *ERG* genes is maintained to achieve sterol homeostasis through binding of Hap1p, Ecm22p and, to a lesser extent, Upc2p to their promotors (Davies and Rine 2006; Vik and Rine 2001). Meanwhile, Rox1p and Mot3p are transcriptional repressors of hypoxic genes, ergosterol uptake genes and genes in the ergosterol biosynthesis pathway, and *ROX1* deletion has been reported to increase the mevalonate level (Liu et al. 2021). In the deficiency of sterols, the Ecm22p-mediated activation of *ERG* genes is repressed by the interaction between Mot3p and Ecm22p, while the content of Upc2p and its binding to the promotors of *ERG* genes is highly strengthened which is responsible for the activation of *ERG* genes under this condition (Davies and Rine 2006; Davies et al. 2005; Vik and Rine 2001). Overexpression of Ecm22p in *S. cerevisiae* was found to enhance the production of ergosterol by upregulation of *ERG* genes except for *ERG4, ERG9* (squalene synthetase) and *ERG28* (ER membrane protein) (Wang et al. 2018). Similarly, overexpression of Upc2-1p (the G888D mutant of *UPC2* (Dong et al. 2020)) improved the production of sterols, such as phytosterols and ergosterols (Ma et al. 2018; Xu et al. 2020). However, overexpression of Upc2-1p in *S. cerevisiae* ∆*ERG5* strain led to decreased ergosta-5,7-dienol and overall sterol contents, which could be explained by the exacerbated redox imbalance in the strain without *ERG5* (Ma et al. 2018).

### Regulation of the accumulation of free sterols

Excessive free sterols in yeast can cause cell damage, and this issue could be solved by esterification of sterols with fatty acids, as well as acetylation and secretion of sterols (Ploier et al. 2015). Are1p and Are2p are homologous proteins of mammalian acyl-coenzyme A (CoA): cholesterol acyl transferase (Acatp) in yeast, which catalyze the esterification of sterols with fatty acids to form SE (Yang et al. 1996). Are1p mainly esterifies intermediates in the post-squalene pathway, such as lanosterol, while Are2p is responsible for the esterification of the final product ergosterol (Jensen-Pergakes et al. 2001). Overexpression of Are2p had no effect on the accumulation of early sterols (such as lanosterol), but enhanced the esterification of ergosterol (Polakowski et al. 1999). In addition, overexpression of Are2p in *S. cerevisiae* led to increased production of ergosterol (He et al. 2007). However, in the 7-DHC-producing *S. cerevisiae*, the deletion of *ARE1* and *ARE2* improved the proportion of 7-dehydrodemosterol which is the direct precursor of 7-DHC, leading to increased 7-DHC production (Hans-Peter et al. 2021). These findings indicate that the production of some sterols such as ergosterol can be improved by enhancing the esterification of free sterols, while biosynthesis of other sterols such as 7-dehydrodemosterol can be increased by weakening sterol esterification and increasing the ratio of free sterols. Interestingly, *ARE1* and *ARE2* deletion seems to be necessary for production of sterols and steroids with limited precursor supply. When constructing the de novo synthesis pathway of sitosterol in *S. cerevisiae*, sitosterol could only be detected after disrupting *ARE1* and *ARE2* to allow for enough free 24-methlyenecholesterol as the substrate of SMT2p, otherwise 24-methlyenecholesterol became inaccessible to SMT2p because of the competitive consumption by highly efficient esterification (Xu et al. 2020). Similarly, the production of hydrocortisone whose precursor is pregnenolone could also be improved by knocking out the gene encoding Atf2p which is responsible for pregnenolone acetylation (Szczebara et al. 2003). When there is a shortage of free sterols in the cells, *S. cerevisiae* improves the supply of free sterols through SE hydrolysis catalyzed by SE hydrolases including Yeh1p, Yeh2p and Tgl1p (Koffel et al. 2005). Tgl1p overproduction was shown to increase the content of ergosta-5-eneol, leading to increased production of pregnenolone and progesterone in *S. cerevisiae* (Duport et al. 2003).

### Regulation of lipid metabolism

The complex interactions among lipids metabolism pathways suggest that the synthesis of sterols and steroids might be affected by regulating lipid metabolism. Regulation of lipid metabolism via engineering ER and LDs was reported to improve the production of sterols (Arendt et al. 2017; Fei et al. 2008). In phospholipid synthesis pathway, diacylglycerol (DAG) is formed from phosphatidic acid (PA) which is one of the membrane lipids under catalysis of phosphatidic acid phosphatase (Pah1p), and the deletion of *PAH1* led to PA accumulation which caused the drastic proliferation of the outer nuclear membrane and the ER (Arendt et al. 2017; Nohturfft and Zhang 2009). The expansion of ER has been successfully used for the functional overproduction of ER-localized proteins which may be beneficial to the production of corresponding metabolites (Arendt et al. 2017). Disruption of *PAH1* boosted the contents of ergosterol and its esterified form in *S. cerevisiae* (Park et al. 2015). However, because DAG is the precursor for TAG which is the main component of LDs (Sorger and Daum 2003), knockout of *PAH1* caused a decrease of the TAG content in yeast, resulting in a decline of the LDs number (Adeyo et al. 2011). LDs are intracellular storage compartments for neutral lipids, and the enhanced formation of LDs through overexpression of Dga1p (TAG synthase) resulted in a 250-fold increase of squalene production (Wei et al. 2018). Similarly, overexpression of *PAH1* and *DGA1* enhanced the production of TAG and lycopene by increasing the storage capacity of LDs (Ma et al. 2019). These results indicate an important role of LDs in the production of lipid-soluble compounds including sterols or steroids. In addition, Nem1p (nuclear envelope morphology-related protein) and Fld1p (seipin in yeast) together play a crucial role in recruiting proteins, including Yft2p (member of the highly conserved FIT family of proteins involved in triglyceride droplet biosynthesis), Pex30p (peroxisome related protein), Pet10p (perilipin), and Erg6p, to the ER subdomains where LDs biogenesis occurs (Choudhary et al. 2020; Choudhary and Schneiter 2020). After knocking out *FLD1* or *NEM1*, giant size of lipid droplets or clusters of small LDs were formed in yeast (Fei et al. 2008; Zhu et al. 2015) and increase of the neutral lipid content was also found, exemplified by the 70% increase in SE synthesis (Fei et al. 2008). Deletion of *FLD1* or *NEM1* in 7-DHC-producing yeast strains increased the production of 7-DHC by 15.7% and 48.3%, respectively (Guo et al. 2018).

## De novo synthesis of typical sterols and steroids using engineered yeast strains

### Sterols

Sterols, including cholesterol from animals, ergosterol from fungi, campesterol and phytosterols from plants, are generally used as key precursors for production of vitamin D, steroid intermediates, and steroid hormone drugs (Fernandes et al. 2003). Great progresses have been made in the de novo synthesis of sterols in yeast by metabolic engineering (Table 1). Advances in production of two typical sterols, 7-dehydrocholesterol and campesterol, are introduced below.

**Table 1:** De novo synthesis of typical sterols in yeast

Products	Strain	Approach			Cultivation mode	Yield/titer/content	References
		Genes deleted	Genes introduced	Genes overexpressed			
Ergosterol	*S. cerevisiae*	–	–	*UPC2*	Flask fermentation	11.91 mg/g	(Ma et al. 2018)
Ergosterol	*S. cerevisiae*	–	–	*ECM22*	5-L bioreactor	32.7 mg/g	(Wang et al. 2018)
Ergosterol	*S. cerevisiae*	–	–	*ARE2*, *ERG4*	5-L bioreactor	1707 mg/L	(He et al. 2007)
Campesterol	*S. cerevisiae*	*ERG5*	*Mo∆7SR*	–	–	–	(Zhang et al. 2007)
Campesterol	*S. cerevisiae*	*ERG5*	*ArDWF1*	–	–	–	(Tsukagoshi et al. 2016)
Campesterol	*S. cerevisiae*	–	*DWF1 /5/7*	All the MVA pathway genes,* UPC2*	Flask fermentation	40 mg/L	(Xu et al. 2020)
Campesterol	*Y. lipolytica*	*ERG5*	*DHCR7*	–	5-L bioreactor	453 ± 24.7 mg/L	(Du et al. 2016)
Campesterol	*Y. lipolytica*	*ERG5*, *MFE1*, *PEX10*	*DHCR7*	*DHCR7*	5-L bioreactor	837 mg/L	(Qian et al. 2020)
Campesterol	*Y. lipolytica*	*ERG5*	*DHCR7*	*POX2*	5-L bioreactor	942 mg/L	(Zhang et al. 2017)
Cholesterol	*S. cerevisiae*	*ERG5*, *ERG6*	*DHCR7*, *DHCR24*	–	Flask fermentation	1 mg/g dry cell weight	(Souza et al. 2011)
Cholesterol	*S. cerevisiae*	*ERG6*, *ATF2*	*DHCR7*, *DHCR24*	*ERG20*, *ERG9*, *ERG1*	Flask fermentation	16 mg/L	(Cheng et al. 2021)
Cholesterol	*Pichia pastoris*	*ERG5*, *ERG6*	*DHCR7*, *DHCR24*	–	–	–	(Hirz et al. 2013)
7-DHC	*S. cerevisiae*	*ERG5*, *ERG6*	*DHCR24*, *ERG2*, *ERG3*	*ERG1*, *ERG11*, *tHMG1*	–	–	(Lang and Veen 2006)
7-DHC	*S. cerevisiae*	*ERG5*	*DHCR24*, *ACS*, *ACL*	*tHMG1*, *ADH2*, *ALD6*	5-L bioreactor	44.49 ± 9.63 mg/L	(Su et al. 2015)
7-DHC	*S. cerevisiae*	*ERG5*, *ERG6*, *NEM1*	*Gg_DHCR24*	All the MVA pathway genes, *Gg_DHCR24*	5‐L bioreactor	1.07 g/L	(Guo et al. 2018)
Ergosta-5,7-dien-3β-ol	*S. cerevisiae*	*ERG5*		*HMG1*, *ERG1*, *ERG11*	Flask fermentation	4.12 mg/g dry cell weight	(Ma et al. 2018)
22-Hydroxycampest-4-en-3-one	*S. cerevisiae*	*ARE1*, *AER2*, *ERG4*	*DWF1/5/7*, *CYP90A1*, *CYP90B1*	*ERG12*, *ERG13*, *ERG8*, *ERG19*	Flask fermentation	3.63 mg/L	(Xu et al. 2020)
β-Sitosterol	*S. cerevisiae*	*ARE1*, *ARE2*, *ERG4*	*DWF1/5/7*, *SMT2*	*ERG12*, *ERG13*, *ERG8*, *ERG19*	Flask fermentation	2 mg/L	(Xu et al. 2020)
24-Methylenecholesterol	*S. cerevisiae*	*ERG4*, *ERG5*	*StDWF5*	–	–	–	(Sawai et al. 2014)
Desmosterol	*S. cerevisiae*	*ERG6*	*StDWF5*	–	–	–	(Sawai et al. 2014)
22(*R*)-Hydroxycholesterol	*S. cerevisiae*	*ERG5, ERG6*	*PpCYP90B27*	–	–	–	(Yin et al. 2018)

*CYP90A1* encoding C3-oxidase; *CYP90B1* encoding C22-hydroxylase; *StDWF5* encoding sterol Δ^7^ reductase; *PpCYP90B27* encoding steroid C22 hydroxylase

#### 7-Dehydrocholesterol

De novo synthesis of 7-dehydrocholesterol in yeast was reported in 2006. By deletion of the intrinsic genes *ERG5* and *ERG6* and overexpression of *tHMG1, ERG1* and *ERG11*, together with the introduction of Erg2p, Erg3p and DHCR24p from mice (*M. musculus*), a 7-DHC-producing *S. cerevisiae* was successfully constructed (Lang and Veen 2006). In 2011, a stable yeast strain RH6826 constructed by deletion of *ERG5* and *ERG6* and introduction of *DHCR24* from *Danio rerio* produced 7-DHC as the main free sterol (86%) (Souza et al. 2011). The production of 7-DHC in *S. cerevisiae* was then improved by increasing the supply of acetyl-CoA and alleviating redox imbalance. *ADH2*, *ALD6*, *ACS* and *ACL* were overexpressed to strengthen the supply of acetyl-CoA in cytosol, leading to 85.44% improvement in the 7-DHC yield. After *NOX* and *AOX1* were introduced to alleviate redox imbalance caused by the deletion of *ERG5*, the production of 7-DHC was further improved by 74.4%, reaching 44.49 ± 9.63 mg/L in fed-batch fermentation (Su et al. 2015). Recently, the highest ever reported 7-DHC production of 1.07 g/L was achieved in *S. cerevisiae* strain constructed from CEN.PK2‐1D expressing *Gg_DHCR24* from *G. gallus* by using a combinatorial engineering strategy. All the functional genes in MVA pathway, including one copy each of *ERG10*, *ERG13*, *ERG12*, *ERG8*, *ERG19*, *IDI1*, and *ERG20* as well as three copies of *tHMG1*, were overexpressed, the competitive branches *ERG5* and *ERG6* were deleted, the expression of P_*GAL1*_-driven *Gg_DHCR24* was improved by knocking out *GAL7,10,1* and doubling the copy number, and *NEM1* involved in lipid metabolism was also knocked out (Guo et al. 2018).

#### Campesterol

The de novo synthesis of campesterol in yeast was first reported as early as 1998, by disruption of *ERG5* and introduction of the *Arabidopsis thaliana* Δ^7^-reductase(Δ^7^REDp) in *S. cerevisiae*, which was then used as the precursor for production of pregnenolone and progesterone (Duport et al. 1998). Since then, campesterol-synthesizing yeast strains were used as the starting strains for production of other sterols and steroids, such as (22S)-22-hydroxycampest-4-en-3-one (Xu et al. 2020) and hydrocortisone (Szczebara et al. 2003). Besides, enzymes responsible for the synthesis of campesterol have been found in various organisms, such as MoΔ7SRp (sterol Δ^7^ reductase from the filamentous fungus *Mortierella alpina* 1S-4) (Zhang et al. 2007), ArDWF1p (oxidoreductase from *A. thaliana*) and DHCR7p (dehydrocholesterol 7-reductase from *D. rerio, Rattus norvegicus, Oryza saliva and X. laevis*) (Du et al. 2016; Souza et al. 2011). Recently, campesterol high-producing *Y. lipolytica* strains were constructed by deletion of *ERG5* and constitutive expression of the codon-optimized *DHCR7* from *X. laevis*, with a campesterol production of 453 ± 24.7 mg/L in high cell density fed-batch fermentation using sunflower seed oil as the carbon source (Du et al. 2016). The yield of campesterol was further improved to 942 mg/L by replacing the *X. laevis DHCR7* with the *DHCR7* gene from *D. rerio* (*DHCR7_Dr*) and overexpressing of Pox2p (Zhang et al. 2017). In another recent study, 837 mg/L of campesterol was produced by regulation of lipid content in *Y. lipolytica* via blocking the gene of multifunctional β-oxidation protein (*MFE1*) (Qian et al. 2020).

### Steroids

The de novo synthesis of steroids received wide attention due to their versatile functions and huge market demands. As early as 1998, the de novo synthesis of pregnenolone and progesterone was achieved in *S. cerevisiae* (Duport et al. 1998). Then, hydrocortisone which was the important intermediate for the synthesis of steroid drugs with potent anti-inflammatory, abortive, or antiproliferative effects was synthesized from progesterone in 2003 (Szczebara et al. 2003). These works opened the door for steroids fermentation from simple carbon source using engineered yeast (Table 2). Below are the progresses in de novo biosynthesis of two typical steroids.

**Table 2:** De novo synthesis of typical steroids in yeast

Products	Strain	Approach			Cultivation mode	Yield/titer/content	References
		Genes deleted	Genes introduced	Genes overexpressed			
Pregnenolone	*S. cerevisiae*	*ERG5*	*ADR*, *ADX*, *P450scc*, *∆7RED*	–	High-density culture	60 mg/L	(Duport et al. 1998)
Pregnenolone	*S. cerevisiae*	*ERG5*	*Δ7RED*, *matADR*, *matADX*, *CYP11A1*	–	High-density culture	2.9 ± 0.5 mg L^−1^*A*_600_ units	(Duport et al. 2003)
Pregnenolone	*Y. lipolytica*	*ERG5*	*CYP11A1*, *ADR*, *ADX*, *DHCR7*	–	5 L bioreactor	78.0 mg/L	(Zhang et al. 2019)
Progesterone	*S. cerevisiae*	*ERG5*	*ADR*, *ADX*, *P450scc*, *∆7RED*, *3β-HSD*	–	High-density culture	60 mg/L	(Duport et al. 1998)
Hydrocortisone	*S. cerevisiae*	*ATF2*, *GCY1*, *YPR1*	*∆7RED*, *ARH1*, *CYP11A1*, *NCP1*, *CYP21A1*, *matADR*, *matADX*, *CYP17A1*, *CYP11B1*, *3β-HSD*	*ARH1*, *CYP21A1*	High-density culture	11.5 mg/L	(Szczebara et al. 2003)
Diosgenin	*S. cerevisiae*	*ERG6*, *ATF2*	*DrDHCR7*, *DrDHCR7*, *DzinCYP90G6*, *VvCPR*, *VcCYP94N1*	*ERG20*, *ERG9*, *ERG1*	Flask fermentation	10 mg/L	(Cheng et al. 2021)
Diosgenin	*S. cerevisiae*	*ERG5*, *ERG6*	*PpCYP90G4*, *PpCYP94D108* or *TfCYP90B50*, *TfCYP82J17*, *CPR*	–	–	–	(Christ et al. 2019)

*matADR* mature form *ADR; matADX* mature form *ADR*; *3β-HSD* encoding 3β-hydroxy steroid dehydrogenase/isomerase; *GCY1*/*YPR1* encoding aldo-keto reductases; *ARH1* encoding ADR-related homolog; *NCP1* encoding NADPH P450 reductase; *CYP21A1* encoding 21-steroid hydroxylase; CYP17A1 encoding 17α-steroid hydroxylase; *CYP11B1* encoding 11β-steroid hydroxylase; *VvCPR CPR* from *Vitis vinifera*

#### Pregnenolone

De novo biosynthesis of pregnenolone in engineered yeast was achieved based on the synthesis of campesterol, which is the direct precursor of pregnenolone. Pregnenolone-producing *S. cerevisiae* was first constructed by disruption of *ERG5*, introduction of *A. thaliana* Δ^7^-reductase together with bovine side chain cleavage cytochrome P450 (P450scc), adrenodoxin reductase (Adrp), and adrenodoxin (Adxp), and the yield was 60 mg/L (Duport et al. 1998). The P450-catalyzed reaction was found as the first and limiting step in the synthesis of steroids (Auchus and Miller 2015). By combinatorial screening of P450scc components pairing sources and regulation of the expression level by promoter optimization of *mCYP11A1*, 78.0 mg/L of pregnenolone was produced in engineered *Y. lipolytica* (Zhang et al. 2019).

#### Diosgenin

Thanks to the construction of the stable cholesterol-producing *S. cerevisiae* strain RH6829 (Souza et al. 2011), the de novo synthesis of steroids using cholesterol as the direct precursor has made great progresses. Diosgenin, which is an important intermediate for the production of steroidal hormones, is one of the typical steroids that were de novo synthesized in RH6829 (Cheng et al. 2021; Fernandes et al. 2003). The biosynthesis pathway of diosgenin was extended from cholesterol by introducing CYP pairs, *PpCYP90G4* (cholesterol 16,22-dihydroxylase)*-PpCYP94D108* (cholesterol 22-monohydroxylase) or *TfCYP90B50* (cholesterol 16,22-dihydroxylase)*- TfCYP82J17* (cholesterol 22-monohydroxylase) from *Nicotiana benthamiana*, together with an *Arabidopsis* CYP reductase (CPR) (Christ et al. 2019). In 2021, an engineered yeast strain producing diosgenin was constructed according to the newly revealed diosgenin biosynthetic pathway in *Dioscorea zingiberensis* whose rhizomes can accumulate around 2–16% diosgenin (Cheng et al. 2021; Huang et al. 2008). The highest ever reported diosgenin production of 10 mg/L in yeast was achieved by co-expression of *DzinCYP90G6* (steroid C-16,22-dihydroxylase) from *D. zingiberensis* and *VcCYP94N1* (steroid C-26 hydroxylase) from *Veratrum californicum* in the cholesterol-producing *S. cerevisiae* strain DG-Cho constructed by deleting *ATF2* and *ERG6*, introducing the *DHCR7* and *DHCR24* genes from *D. rerio*, and overexpressing *ERG1*, *ERG20* and *ERG9* (Cheng et al. 2021).

## Challenges and future perspectives

Although the de novo synthesis of many sterols and steroids has been achieved in yeast, there are still lots of highly valuable steroids whose biosynthesis from simple carbon sources remains to be explored, such as androstenedione (AD), testosterone (TS), spirostane-type saponins, brassinosteroids, prednisone and dexamethasone (Fig. 1). The construction of diosgenin biosynthesis pathway in *S. cerevisiae* as mentioned in this review indicates that scrutinizing the genome of eukaryotes possessing the native targeted sterols or steroids pathway and integrating these genes into engineered yeast is a promising strategy for achieving de novo synthesis of the yet-to-explore sterols or steroids (Huang et al. 2008).

For the sterols and steroids that have been successfully produced in yeast, their yields need to be further improved by addressing the remaining challenges, such as the rate-limiting steps in the post-squalene pathway leading to the large accumulation of squalene (Jandrositz et al. 1991), the poor adaptation of heterogenous enzymes especially CYP P450 enzymes for the biosynthesis of steroids causing limited efficiency of the hydroxylation and side chain cleavage reactions (Duport et al. 1998; Zhang et al. 2019), the damage to sterol homeostasis because of the excess free sterols (Ploier et al. 2015) and the limitation of storage room in yeast (Wei et al. 2018). For relieving the metabolic bottleneck caused by rate-limiting enzymes, typical strategies include gene overexpression (Veen et al. 2003) and screening the enzymes from various sources (Guo et al. 2018), but these strategies did not fully address this issue. Protein engineering may be a good solution. However, it encounters difficulties due to the lack of proper high-throughput screening methods and understanding on the protein structure–function relationship. Recent advances in protein structure prediction tools, like RoseTTAFold and Alphafold, provide insight into protein function independent of experimentally determined structures (Baek et al. 2021; Jumper et al. 2021), and are expected to facilitate protein redesign for improved catalytic performance. For the P450-catalyzed reactions, the efficiency may be enhanced by regulation of the P450 expression level by promoter engineering, improving its catalytic activity by protein engineering, and promoting electron transfer by fusion expression of P450 and CPR (Jiang et al. 2021). For reconstruction of sterol homeostasis, strategies including relieving the toxicity of excess sterols by overexpressing Are2p (He et al. 2007) and enlargement of LDs by deleting *FLD1* or *NEM1* (Guo et al. 2018) were employed to regulate lipid metabolism and efficiently enhanced the de novo synthesis of sterols or steroids. Many other genes that are involved in LDs biogenesis from specialized ER subdomains such as *LRO1* (triacylglycerol synthase), *DGA1*, *PEX30* and *YFT2* (Choudhary and Schneiter 2020), could also be included as regulation targets in the future.

The competition between heterologous and endogenous sterol metabolism is another limiting factor to be taken into account as revealed in the de novo synthesis of β-sitosterol in *S. cerevisiae* (Xu et al. 2020). Systems biology coupling with synthetic biology and evolutionary engineering may be a prospective approach to optimize the performance of the engineered sterols/steroids-producing yeast by driving the cycle of design–build–test–learn (Campbell et al. 2017). Considering the complex metabolic network for sterols and steroids biosynthesis, machine learning is a promising approach for optimizing the metabolic flux (Kim et al. 2020). In addition, computation-guided design of artificial synthetic pathways with lower complexity that is orthologous to the native sterol metabolism represents an attractive future direction.

In conclusion, biosynthesis of sterols and steroids using yeast cell factories would be a prospective production route of these increasingly sought-after drug intermediates after addressing the remaining challenges.

## Data Availability

Not applicable.

## Abbreviations

7-DHC: 7-Dehydrocholesterol
GRAS: Generally regarded as safe
BD: Boldenone
RSS: Cortexolone
TS: Testosterone
DHEA: Dehydroepiandrosterone
ADD: Androst-1,4-ene-3,17-dione
9α-OH-AD: 9α-Hydroxyandrost-4-en-3,17-dione
AD: Androstenedione
ER: Endoplasmic reticulum
LDs: Lipid droplets
SE: Steryl esters
TAG: Triacylglycerols
SRE: Sterol regulatory element
ABC: ATP-binding cassette
BST: Biochemical Systems Theory
SL-E: Sphingolipid–ergosterol
PM: Plasma membrane
MVA: Mevalonate
DAG: Diacylglycerol
PA: Phosphatidic acid
HMG-CoA: 3-Hydroxy-3-methylglutaryl-CoA
FFA: Free fatty acids
CDP-DAG: Cytidine diphosphate-diacylglycerol
PI: Phosphatidylinositol
PS: Phosphatidylserine
PE: Phosphatidylethanolamine
PC: Phosphatidylcholine
